# Childbirth preferences and related fears - comparison between Norway and Israel

**DOI:** 10.1186/s12884-018-1997-5

**Published:** 2018-09-05

**Authors:** Heidi Preis, Yael Benyamini, Malin Eberhard-Gran, Susan Garthus-Niegel

**Affiliations:** 1 Bob Shapell School of Social Work, Tel Aviv University, Tel Aviv, Israel; 2 Department of Child Health, Norwegian Institute of Public Health, Oslo, Norway; 3 Health Services Research Unit, Akershus University Hospital, Lørenskog, Norway; 4 Institute of Clinical Medicine, University of Oslo, Oslo, Norway; 5 Department of Psychotherapy and Psychosomatic Medicine, Faculty of Medicine of the Technische Universität Dresden, Germany, Dresden, Germany

**Keywords:** Fear of childbirth, Cultural comparison, Childbirth preferences, Cesarean section, Epidural analgesia, W-DEQ, Confirmatory factor analysis

## Abstract

**Background:**

Fear of childbirth (FOC) could have significant impact on women’s childbearing choices and experience. Culture affects the way women conceptualize childbirth, influencing the fears and expectations they may hold in relation to it. In the current study, we examined differences in childbirth preferences of cesarean section and use of epidural analgesia between Norwegian and Israeli pregnant women. Later, we used the Norwegian six-factor solution of the widely-used Wijma Delivery Expectancy Questionnaire (W-DEQ-A) to compare levels of the different FOC factors. Finally, we investigated differences in the associations between FOC and childbirth preferences between the two countries.

**Methods:**

Secondary analysis of two large surveys. Women from Israel (*n* = 490) and Norway (*n* = 2918) were recruited during prenatal check-ups in community clinics and a university hospital. At around 32 weeks of gestation, all participants filled out questionnaires, including the W-DEQ-A. Statistical analysis included exploratory factor analysis, confirmatory factor analysis, M/ANOVA, Spearman’s Rho and Fisher’s *Z* tests for the significance of the difference between independent correlations.

**Results:**

The Norwegian six-factor solution of the W-DEQ fit well with the Israeli data. Norwegian women were more concerned about loneliness, feeling less self-efficacy, negatively appraising birth, and lacking positive anticipation. Israeli women were more concerned about negative outcomes for the child and experienced greater general fear and fear of pain. Norwegian women preferred more cesarean sections compared to Israeli women, who preferred more epidural use than Norwegians. FOC factors were more strongly related to childbirth preferences among Norwegians.

**Conclusions:**

Cultural differences between Israel and Norway are reflected by the differences seen in the levels of fear reported across the six factors. In Israel, birth culture is very medicalized, motherhood is highly revered, and there is an emphasis on having “perfect babies”. In contrast, Norwegian women have fewer children, and birth is considered more natural. This could explain why Israeli women were more concerned that their child might be harmed during birth, while Norwegian women were more concerned with the physical and emotional expectations of birth.

**Electronic supplementary material:**

The online version of this article (10.1186/s12884-018-1997-5) contains supplementary material, which is available to authorized users.

## Background

Childbirth is one of the most notable events in a woman’s life. It transitions women to motherhood and has substantial physical as well as emotional impacts. In many Western countries, the childbirth process has been medicalized and childbirth is thus mostly performed in hospitals, where it is managed by medical professionals with the use of technology [1]. Technological births such as the cesarean section (CS) and use of epidural analgesia (EA) may be lifesaving and alleviate women’s pain during birth, but they may also have adverse effects on mothers’ health and well-being [2, 3].

Women often take part in the decision-making process regarding their deliveries and it is important to understand the factors that influence women’s childbirth preferences. Cultural norms regarding motherhood and birth shape women’s perceptions regarding what birth is and how it should be managed. Several studies have found differences between cultures in terms of women’s preferences regarding CS [4, 5], EA [6, 7], and levels of fear of childbirth (FOC) [8–10]. In addition, preliminary findings suggest that the known association between FOC and preferences for CS [11, 12] may be culture-bound [5, 13]. In the current study, we wished to compare women’s childbirth preferences and the way they relate to FOC between two Western countries - Norway and Israel.

### Birth culture in Norway and Israel

In Norway - and Scandinavia as a whole - there is a strong norm of pregnancy and childbirth being *natural processes*. Antenatal care is primarily midwife-led and patient-centred with a focus on shared decision-making and on avoiding unnecessary examinations [14]. For example, there is usually only one regular ultrasound scan during the entire pregnancy, and except for several blood tests early in pregnancy, measurement of fundal height and blood pressure, not many medical tests are conducted in healthy women with low-risk pregnancies [14]. The CS rate is relatively low compared to other Western countries (with 6.6% elective and 10.5% emergency CS) [15]. Home birth rates are close to 3% [16], and for low-risk pregnancies, the main birth attendant is a midwife [14]. Another relevant cultural norm relates to individualism and female autonomy. As Norwegian women’s roles in modern society have changed over the last 50 years, so have their reproductive patterns. Norwegian women feel that they have the individual freedom to plan their reproduction; therefore, they increasingly devote less of their lives to pregnancy and childcare [17]. Consequently, in the last century, the number of children born per woman has steadily declined. In 2015, the fertility rate for women in Norway was 1.71 [18].

Israel, conversely, is more pro-natal and patriarchal [19] and has the highest fertility rate (3.1) among the Organization for Economic Co-operation and Development (OECD) countries (average in the OECD is 1.7) [18]. Israeli women are expected to be mothers, and birth has been referred to as a “national mission” aimed at increasing the Jewish population, which was reduced during World War II [19, 20]. Childlessness is highly stigmatized; therefore, having a child is in practice not a choice but an obligation. Israel is one of the leading nations in reproductive technologies and provides subsidised fertility treatments for up to two children [19]. Moreover, there is pressure to have “perfect” babies [21], which coincides with the excessive medicalization of women’s reproductive health [19, 20, 22]. Women are offered numerous antenatal ultrasound scans and various tests over the course of the pregnancy to ensure the healthy development of the baby. Practices such as preimplantation genetic testing or abortions for unspecified foetal anomalies are legal and not uncommon compared to other countries [23]. In some hospitals, the use of EA reaches up to 90% among nulliparae [24], and overall CS rates are 19.0% [25], exceeding the 10–15% rates recommended by the World Health Organization [26]. Home birth is discouraged (less than 1% rate) [20], and while midwives do attend uncomplicated births, obstetricians oversee them.

### Conceptualization of fear of childbirth

Being concerned about birth is a normal and prevalent emotion. While some women may show little FOC; others might experience moderate, adaptive, and harmless levels; and, for some women, this fear may be a dominant emotion during pregnancy and may seriously influence their daily lives [27]. However, there is no single, agreed-upon definition of FOC (for a detailed discussion, see the systematic review by Nilsson et al., [10]). The content of what women fear may differ among individuals and may involve different domains of the birth process, such as: fear that labor will be accompanied by intolerable pain, fear of not being competent to give birth, or concerns about the health of the baby [28–30].

One of the most widely-used instruments to measure prenatal FOC is the Wijma Delivery Expectancy Questionnaire (W-DEQ) version A [31]. The scale has 33 items and may be used as a dichotomous scale (with varying cut-off scores to denote severe FOC (see for example [9, 30]) or as a continuous variable (see for example [8]). The scale was originally used as a unidimensional instrument measuring FOC [31]. Recently, researchers have suggested that there may be disadvantages to constructing fear by totalling scores on the W-DEQ [32]. Indeed, when examining the scale, it is noticeable that it reflects not only different fears women may have regarding birth but also other fears and expectations regarding the birth experience. This observation has been empirically supported by several studies that extracted separate factors from the scale [8, 30, 33, 34]. These factors operationalize the conceptual dimensions of women’s fears. Although there is no clear and agreed upon definition of FOC, a similar structure found in different cultures suggest women view childbirth along similar lines. Some of the common dimensions that were identified within the W-DEQ are: general or pain fear, concerns regarding isolation or loneliness during birth, lack of positive anticipation, and concerns for the child’s health [33]. In a large Norwegian study by Garthus-Niegel et al. [35], the number of items in the scale was reduced to 25 and six different factors of the W-DEQ were identified using exploratory factor analysis (EFA) and confirmatory factor analysis (CFA). Relating to the different dimensions of FOC may improve the assessment of their correlates, as some studies have found differences in the way the factors associate with childbirth preferences [30].

### The current study

Though there are similarities between Norway and Israel (similar CS rates, midwives as main birth attendants, low home birth rates) they clearly have different birth cultures and normative expectations regarding motherhood, which might have implications for birth preferences and FOC. Therefore, the aim of this paper was: (a) to compare childbirth preferences of CS and EA between the two countries; (b) to compare FOC levels as measured by the W-DEQ between expectant mothers in Israel and Norway; and, (c) to investigate how FOC factors are related to the women’s preferences for CS and EA in the respective countries.

## Methods

### Procedure and setting

The current study was a secondary analysis of two large studies that were conducted independently over a period of five years in Israel [22] and in Norway [35]. Recruitment for the Norwegian study took place between November 2008 and April 2010 at the Akershus University Hospital near Oslo. Recruitment for the Israeli study took place between May 2012 and December 2013 at women’s health centers in a large metropolitan area in central Israel. In both countries, after receiving an explanation about the study, women provided their written consent and then were asked to fill out the questionnaire. Eligible women were those able to complete the questionnaires in Norwegian or Hebrew, respectively, and who had the option to have a vaginal delivery [22, 36].

### Participants

The study sample included 2918 Norwegian and 490 Israeli pregnant women. Mean age when completing the questionnaire was 31.1 (±4.8) for the Norwegian sample and 32.2 (±4.6) for the Israeli sample. Mean gestational week when filling in the questionnaire was 32.6 (±0.5) for the Norwegian sample and 31.8 (±7.2) for the Israeli sample. The Norwegian sample had 49.8% (*n* = 1463) nulliparae while the Israeli sample had 39.0% (*n* = 191) nulliparae. Details on each sample and comparison are presented in Additional file 1.

### Measures

#### Childbirth preferences

Women were asked how likely they were to choose or how much they would prefer to have a CS and to use EA during labor and delivery. In the Norwegian study questionnaire, women were presented with the statements: “If I could choose, I would rather deliver/prefer to deliver by CS” and “I would rather have epidural anesthesia”, with the response options ranging from 4 = fully disagree, 3 = disagree, 2 = agree, to 1 = fully agree. In the Israeli study questionnaire, women were asked whether they had considered: choosing to deliver by CS / asking to have epidural anesthesia, with the answers for each question ranging from 0 = did not consider it at any stage, 1 = considered but no chance that I will make that choice, 2 = small chance, 3 = medium chance, 4 = high chance, I have decided to make that choice. To make the two scales comparable, the Norwegian data were reverse scored and the Israeli 0 and 1 scores were combined so that the answers in both samples ranged from 1 to 4, with higher scores indicating greater agreement with the preference. In addition, we also coded a dichotomous scale of preference with scores 1–2 = not likely to make that choice and 3–4 = likely to make that choice.

*Fear of Childbirth* (FOC) was assessed using the *Wijma Delivery Expectancy Questionnaire* (W-DEQ-A; [31]), a widely-used tool to prenatally assess various aspects of FOC. The scale had been previously translated from the English version (forward and back translation) and validated in Norwegian [37] and in Hebrew [22]. The scale consists of 33 items that are scored from 0 to 5. In both countries, women completed an equivalent version of the full scale. Factor scores were calculated by averaging items, after reversing relevant items so that all high scores indicated greater FOC.

### Statistical analysis

Analyses were conducted using SPSS version 24.0 [38] and AMOS 22 [39]. The dichotomous preference scores were used for comparing rates between the countries. In order to perform EFA on the W-DEQ Israeli data, we initially conducted parallel analysis, a more accurate method for determining the number of factors in a set of items than the commonly used criterion of eigenvalue greater than 1 [40]. Thereafter, we conducted EFA with varimax rotation while forcing the number of resulting factors. Following this step, the Norwegian structure of the W-DEQ was examined on the Israeli data using CFA. We used widely acceptable fit indices, Comparative Fit Index (CFI) and Root Mean Squared Error of Approximation (RMSEA), to estimate the goodness of fit of the CFA. CFI measures how well a model fits the data, with 1.00 being perfect fit and 0.90 or above considered an acceptable fit. RMSEA measures the discrepancy between the estimated model and the data, with values equal or less than 0.06 indicating a good fit. Missing data on single W-DEQ items tested in the Israeli sample ranged from 0.2 to 5.9%, and expectation-maximization technique was used to replace the missing values. Because several studies have found parity-based differences between women, we stratified our analyses accordingly. Comparisons on FOC factors and preferences were done through univariate and multivariate analyses of variance (M/ANOVA) testing for the effect of country, parity, and the interaction between them. Significance and effects sizes differences between countries on FOC factors were calculated using independent *t* tests and Cohen’s *d*. The associations between FOC and childbirth preferences were calculated on the basis of the continuous preferences scores using Spearman’s Rho coefficients. These correlations were then compared between the countries using Fisher’s *Z* tests for the significance of the difference. Confidence levels were set at 95% and a *p*-value < 0.05 (two-tailed) was interpreted as statistically significant.

## Results

### Comparing childbirth preferences between the two countries

There was a great difference in childbirth preferences between the two countries. As seen in Table 1, opposite preferences regarding EA were revealed: In Norway, close to three quarters of the women preferred *not* to receive EA, while in Israel, three quarters of the women preferred to receive EA. With regards to CS, twice as many Norwegians (9.6%) would have preferred CS compared to Israeli women (5.0%). There was no significant country by parity interaction.

**Table 1:** Comparison of childbirth preferences

	Preference for epidural analgesia n (%)			Preference for cesarean section n (%)		
	Prefer	Does not prefer	χ^2^	Prefer	Does not prefer	χ^2^
Norway	659 (22.8)	2234 (77.2)	505.55 *p* < 0.001	287 (9.9)	2618 (90.1)	11.53 *p* < 0.001
Israel	334 (74.3)	119 (25.7)		23 (5.0)	440 (95.0)	

Norwegian questions: “If I could choose, I would rather deliver/prefer to deliver by CS?” and “I would rather have epidural anesthesia?” Israeli questions: “Have you considered choosing to deliver by CS?” and “Have you considered choosing to deliver with epidural anesthesia?”

### Establishing a comparable FOC measure

Parallel analysis revealed that there were seven factors in the Israeli on the 33 item W-DEQ data. Thereafter, we conducted EFA with a varimax rotation while forcing seven factors. This analysis yielded a very poor structure solution, with several factors loading on a single item or items with no factors loading on them (Additional file 2). Since the EFA did not produce comprehensible and determinable factors, we decided to test the Norwegian factor structure with the Israeli data. CFA was conducted to examine the validity of the Norwegian, 25-item, six-factor structure [35] in the Israeli data. Table 2 presents the similar factor loadings of the Norwegian and Israeli data on each factor. Fit indices for the six-factor solution suggest an acceptable model fit with the Israeli data: χ^2^ (257) = 02.23, *p* > 0.05 RMSEA = 0.06, CFI = 0.91, thus validating the structure. We also computed the factors’ internal reliabilities and found them to range between α = 0.73 and α = 0.88, similar to the range reported in the Norwegian study [35] (Table 2).

**Table 2:** Factor loadings of the six-factor W-DEQ structure, reliability and distribution

Item no.	Factor/ item	Norway	Israel
		α, *M ± SD*, actual range/ standardized factor loadings	α, *M ± SD*, actual range/ standardized factor loadings
Fear		0.81, 2.59 *±* 0.87, 0–5	0.75,2.64 *±* 0.9, 0–5
6	Afraid	0.72	0.61
12	Tense	0.62	0.54
19	Panic	0.85	0.83
20	Hopelessness	0.73	0.67
24	Pain	0.42	0.53
27	Lose control	0.51	0.28
Negative appraisal		0.87, 1.47 ± 0.97, 0–5	0.84, 1.22 ± 0.92, 0–5
1	Not fantastic	0.63	0.58
13	Not glad	0.84	0.65
14	Not proud	0.84	0.76
18	Not happy	0.86	0.88
Loneliness		0.81, 0.75 ± 0.89, 0–5	0.82, 0.49 ± 0.77, 0–5
3	Lonely	0.74	0.71
7	Deserted	0.84	0.90
15	Abandoned	0.70	0.77
Lack of self-efficacy		0.82, 2.10 ± 0.77, 0–4.86	0.73, 0.75 ± 0.75, 0–4.67
4	Not strong	0.69	0.77
5	Not confident	0.75	0.90
9	Not safe	0.60	0.48
10	Not independent	0.63	0.54
16	Not composed	0.48	0.31
22	No self-confidence	0.73	0.73
26	Not let happen	0.49	0.02
Lack of positive anticipation		0.75, 0.75 ± 0.75, 0–4	0.78, 0.52 ± 0.75, 0–4.67
28	Not joyful	0.62	0.66
29	Not natural	0.87	0.78
30	Not obvious	0.66	0.78
Concerns for the child		0.88, 0.81 ± 1.15, 0–5	0.88, 1.49 ± 1.42, 0–5
32	Fantasies that child will die	0.84	0.97
33	Fantasies that child will be injured	0.93	0.81

Comment: All parameters were significant except for item 26 in the Israeli data

### Comparing FOC factors

Mean levels on the FOC factors by country and parity are presented in Table 3 (total sample central tendencies are presented in Table 2). MANOVA showed significant differences by country (*F*(6,3340) = 51.34, *p* < 0.001, partial *η*^2^ = 0.08) and by parity (*F*(6,3340) = 37.26, *p* < 0.001, partial *η*^2^ = 0.06). The interaction between country and parity was not significant (*F*(6,3340) = 1.53, *p* = 0.16, partial *η*^2^ = 0.00). In both countries, nulliparae showed greater fear in most factors. Overall, Norwegian participants were more concerned than Israeli participants about: having a negative appraisal during delivery, being lonely, and lacking self-efficacy. In addition, Norwegian multiparae were more concerned about not having positive anticipation. On the other hand, Israeli participants were more concerned about having a negative outcome during birth and had higher levels of general and pain fear. Almost all the differences in the factor means between countries were significant, with a small to medium effect size.

**Table 3:** Differences in FOC factors by country and by parity

	Nulliparous				Multiparous			
	Norway *M* (*SD*)	Israel *M* (*SD*)	*t*	Cohen’s *d*	Norway *M* (*SD*)	Israel *M* (*SD*)	*t*	Cohen’s *d*
Fear	2.78 (0.80)	2.92 (0.86)	2.22^*^	−0.17	2.39 (0.90)	2.47 (0.92)	1.21	−0.9
Negative appraisal	1.40 (0.95)	1.17 (0.99)	−3.11^**^	0.24	1.54 (0.98)	1.25 (0.88)	−4.75^***^	0.31
Loneliness	0.76 (0.87)	0.53 (0.80)	−3.64^***^	0.28	0.74 (0.91)	0.47 (0.76)	−5.35^***^	0.32
Lack of self-efficacy	2.21 (0.72)	1.89 (0.76)	−5.74^***^	0.43	1.98 (0.79)	1.74 (0.69)	−5.43^***^	0.32
Lack of positive anticipation	0.83 (0.76)	0.71 (0.88)	−1.73	0.15	0.66 (0.74)	0.39 (0.62)	−6.55^***^	0.40
Concerns for the child	0.78 (1.14)	1.40 (1.38)	5.93^***^	−0.49	0.84 (1.16)	1.55 (1.45)	7.96^***^	−0.54

*p < 0.05, **p < 0.01, ***p < 0.001

### Association of FOC factors and childbirth preferences

The associations between FOC levels and the childbirth preferences differed between the two cultures (Table 4). Overall, among the Norwegian women, there were significant weak to moderate correlations between FOC and preferring a more medicalized birth (CS and EA), while among the Israeli women, all correlations were weak and most were non-significant. Among Norwegian women, all the FOC factors were correlated to preferring EA. Among Israelis, preferring EA was linked only to concerns regarding lack of self-efficacy. Among Norwegian women, all FOC factors were associated with CS preference while among Israeli women, that preference was associated only with the “fear” factor. Many of the correlations differed significantly in size between the two countries (*Z* scores over 1.96). Between-country correlation comparisons stratified by parity showed similar results (not presented).

**Table 4:** Spearman’s Rho correlations between FOC factor levels and childbirth preferences

	Preference for epidural analgesia			Preference for cesarean section		
	Norway	Israel	*Fisher’s Z* ^a^	Norway	Israel	*Fisher’s Z* ^a^
Fear	0.32^***^	0.07	5.21^***^	0.30^***^	0.11^*^	3.97^***^
Negative appraisal	0.20^***^	0.06	2.84^**^	0.21^***^	0.05	3.25^**^
Loneliness	0.17^***^	−0.07	2.02^*^	0.20^***^	0.08	2.44^*^
Lack of self-efficacy	0.31^***^	0.13^**^	3.78^***^	0.29^***^	0.07	4.55^***^
Lack of positive anticipation	0.11^***^	0.01	1.98^*^	0.14^***^	0.05	1.79
Concerns for the child	0.10^***^	0.08	0.40	0.13^***^	0.09	0.80

^*^p < 0.05, ^**^p < 0.01, ^***^p < 0.001

^a^Z for the difference between the correlations in the two countries

## Discussion

Our findings showed both similarities and differences related to the perception of childbirth in Norway and Israel. Women’s preferences regarding CS and EA differed between the two countries. The six-factor structure of W-DEQ version A, reported earlier in the Norwegian sample [35], was validated in this study with data from pregnant women in Israel. In both countries, first-time mothers reported more FOC compared to women who had previously given birth. FOC levels differed by country, with Norwegian women scoring higher on some factors and Israeli women on others. Among Norwegians, FOC levels were related to their childbirth preferences while among Israelis they were not.

These differences reflect the overall more natural birth culture in Norway, compared to the more medicalized practices in Israel. This is seen in women’s preferences: while most women (over 90%) in both countries wished to have a vaginal delivery, about three quarters of the Israeli women preferred EA, while over three quarters of the Norwegian women preferred *not* to have it. These findings are consistent with the much lower EA use in Norway [41] compared to Israel [25]. In Norway, the emphasis is on women’s autonomy, first in choosing to have a child and later in the process of natural labor and delivery [17]. The natural birth discourse begins in pregnancy and focuses more on the woman’s role and her strength to carry it out and less on the possible risks during birth. Unless there is a known risk factor (such as older age), women undergo only basic pregnancy check-ups with a midwife and only one ultrasound scan during the entire pregnancy, which is performed at around 17–19 weeks of gestation [14]. It is not surprising that Norwegian women, who are expected to give birth naturally, are less inclined to plan the use of medical pain relief.

In contrast, in Israel the discourse around pregnancy and childbirth is much more medical. Pregnant women undergo monthly check-ups in an obstetrician’s office, which most often include an ultrasound examination. In addition, extensive blood tests are taken, and elaborate ultrasound scans of all fetal systems are performed by specialists at around 13–17 and 20–23 weeks of gestation. This risk-instilling environment promotes the search for the “perfect baby” [21], which is supported by the medical, legal, and religious establishment in Israel [23]. Childbirth preparation classes and labor room tours provide much information about medical interventions during childbirth, and it is legitimate to plan for and ask about various interventions and ways to ensure maternal and fetal safety. The Ministry of Health recently set guidelines to manage natural birth in hospitals, which denote that natural birth is not the norm [42]. Medical interventions are frequently used, from birth induction (15.2% of births), to EA (42.5% of births) and elective CS (13.8% of all births) to surgically-assisted emergency vaginal (5.5% of births) or CS (5.2% of all births) [25]. Altogether, the focus is more on birth as a painful and risky process and on ensuring that the perfect child is produced [22]. Therefore, it is not surprising that Israeli women, who often view birth pain as a medically needless inconvenience, were more concerned with pain and wished to have EA.

While most women in both samples would not choose delivery by CS, 10% of the Norwegian women were inclined to choose a CS if they could, compared to only 5% of the Israeli women. It is possible that since women in Norway have more autonomy and are more involved in the decision-making process during birth [14], they are more likely to consider asking for a CS. This finding is in concordance with the relatively high known rate of 7.6% CS by maternal request out of all CS in Norway [43] and lower rate of 2.1% in Israel [25]. It is important to note that the higher fertility rates in Israel may also explain these findings [19]. Because of the adverse effect CS could have on future reproduction and birth, women who wish to have more children would rather avoid a CS [44].

The six factors that were identified in the Norwegian sample [35] were also present in the Israeli sample. Thus, despite the differences in the birth culture in these countries, there seems to be a uniform core to FOC dimensions as reflected by the W-DEQ factors. The concurrent exploration of the factors adds richness to the cross-cultural comparison, as it allows researchers to address nuances otherwise overlooked. These factors seem to represent a variety of cognitive expectations and concerns which may be culturally influenced, as in our case. It is possible that the “fear” factor, which was most strongly associated with childbirth preferences, is separable from the rest of the factors that tap into women’s expectations [34]. Therefore, for certain purposes, it may be helpful or sufficient to use the shorter six-item fear factor, as in, for example, studies that want to focus on fear or in settings where a brief screening tool is needed.

While the W-DEQ structure replicated across the two cultures, there were substantial differences between Norwegian and Israeli women in our study in the levels reported on the FOC factors. Norwegian women were significantly more concerned with the factors associated with the subjective birth *experience*: They were concerned about the birth not turning out “as it should be” – natural and joyful, a powerful and empowering experience, during which they would feel strong and confident. They worried about having a negative experience. Compared to them, Israeli women, who are often surrounded by family members or other support (such as a doula) during labor and delivery, were barely concerned that they would feel lonely and deserted. Israeli women were more worried about the birth *process and outcomes* – they were more likely to report fear of the birth in general, of the pain, tension, and possible panic and loss of control. They were also more worried about the health and safety of the baby. This could also be explained by the cultural difference in the number of desired children: If you plan to give birth to only one or two children, as most women in Norway do, you may be more invested in having the ultimate birth experience. On the other hand, when you know you are likely to have three or more children, even if one of these experiences is more difficult, you will also have more chances for a good birth experience.

In Norway, as in other Scandinavian countries, there is much research on and more awareness of FOC [9, 32] and it is more acceptable to request a CS for that reason [45, 46]. Indeed, among Norwegian women, scores on all six factors of W-DEQ were correlated with a greater preference both for EA and CS. Regarding the Israeli sample, previous findings showed that while many Israeli women endorsed medical beliefs about birth and preferred giving birth under medical supervision [22], at the same time most of them also believed that birth is a natural process [47]. This belief coincides with the medical system’s strong discouragement of CS by maternal request, thus creating very low rates of CS with no medical indication [25]. The high usage of EA and low rates of CS by maternal request likely both reflect physicians’ beliefs and preferences and women’s agreement with them and are thus different manifestations of the medicalization of childbirth in Israel. The flip-side of these findings can be seen in the lack of associations between the FOC factors and the Israeli women’s preferences. These correlations were very weak, all significantly lower than the ones found in the Norwegian sample. In countries where the medical system does not encourage a diversity of choices in childbirth [19], it is possible that FOC has less of an effect on women’s preferences.

### Strengths and limitations

Similar to most of the research on FOC, our study is limited by focusing only on Western countries. Its strength lies in comparing two countries that are similarly modern yet of quite different birth cultures, thus uncovering both the common elements and the differences in the possible effects of FOC. Several methodological limitations must be acknowledged. Firstly, translation of the W-DEQ (into Norwegian and Hebrew) might affect the understanding of the items; thus, even with careful translation, differences may have resulted from the wording of the scale or the different expressions of emotions in each culture. To ensure as accurate a translation as possible, we used forward and backward translation, both done from the English version. Secondly, we determined the structure of the Israeli data by conducting CFA of the Norwegian six-factor solution but did not present an autonomous solution. Our study did not aim to uncover the ideal structure for the Israeli data, but rather tested whether the Norwegian structure was adequate so that it could be used as a basis for the cross-national comparison. The six-factor solution presented should be cautiously utilized. We recommend that before applying this model to other cultures, it should be cross-validated and tested and that the WDEQ structure should be confirmed in the specific data. Lastly, the findings were derived from secondary analyses of existing data from two studies that were not planned or executed simultaneously, which may have affected the comparability of the data. Therefore, the differences in childbirth preferences should be interpreted with caution. Nonetheless, both studies were based on large samples from clinical settings, allowing for the possibility to compare country trends in childbirth preferences and their relation to FOC.

## Conclusion

Dimensions of FOC (as measured by the W-DEQ version A factors) were similar in Norway and Israel, yet levels of FOC, birth preferences, and the correlations between the two differed between these cultures. Cross-cultural studies such as this are important, as they uncover and highlight the cultural norms around women’s reproductive health and the ways they affect women’s expectations, fears, and preferences. Childbirth always involves uncertainty, and consequently can raise various concerns. Our findings underscore the importance of acknowledging that different women are afraid of different aspects of childbirth and that this may be partly due to the birth culture that surrounds them. It is important to attend to FOC during pregnancy and provide appropriate support.

## Additional files

Additional file 1: Sample characteristics and comparison. (DOCX 15 kb)

Additional file 2: EFA of the Israeli data. (DOCX 20 kb)

## Abbreviations

CFA: Confirmatory Factor Analysis
CS: Cesarean Section
EA: Epidural Analgesia
EFA: Exploratory Factor Analysis
FOC: Fear of Childbirth
W-DEQ: Wijma Delivery Expectancy Questionnaire
