# Supplementary material for: Childbirth preferences and related fears - comparison between Norway and Israel
Source: BMC Pregnancy Childbirth. 2018 Sep 5;18:362. doi: 10.1186/s12884-018-1997-5 (PMC6126000; doi:10.1186/s12884-018-1997-5)
Supplement: Supplementary file 1 — Sample characteristics and comparison. (DOCX 15 kb) [file 12884_2018_1997_MOESM1_ESM.docx]

**Additional file 1**

**Sample characteristics and comparisons**

|  | **Norway**  **2936 (100)**  **n (%)** | **Israeli**  **490 (100)**  **n (%)** | **χ^2^** |
| --- | --- | --- | --- |
| **Education** |  |  | 4.17^*^ |
| ≤12 years of education | 944 (34.9) | 147 (30.1) |  |
| > 12 years of education | 1762 (65.1) | 341 (69.6) |  |
| **Marital status** |  |  | 1.07 |
| Married/cohabiting | 2,714 (97.3) | 470 (96.5) |  |
| Single/divorced | 74 (2.7) | 17 (3.5) |  |
| **Parity** |  |  | 19.80^***^ |
| Nulliparae | 1,463 (49.8) | 191 (39.0) |  |
| Multiparae | 1473 (50.2) | 299 (61.0) |  |
| **Previous pregnancy loss** |  |  | 3.13 |
| No | 2236 (76.2) | 355 (72.5) |  |
| Yes | 700 (23.8) | 135 (27.5) |  |
| **Fertility treatments** |  |  | 46.58^***^ |
| No | 2823 (96.1) | 436 (89.0) |  |
| Yes | 113 (3.9) | 54 (11.0) |  |
