# Supplementary material for: Childbirth preferences and related fears - comparison between Norway and Israel
Source: BMC Pregnancy Childbirth. 2018 Sep 5;18:362. doi: 10.1186/s12884-018-1997-5 (PMC6126000; doi:10.1186/s12884-018-1997-5)
Supplement: Supplementary file 2 — EFA of the Israeli data. (DOCX 20 kb) [file 12884_2018_1997_MOESM2_ESM.docx]

**Additional file 2**

**EFA of the Israeli data**

|  |  | 1 | 2 | 3 | 4 | 5 | 6 | 7 |
| --- | --- | --- | --- | --- | --- | --- | --- | --- |
| 1 | Not fantastic |  | **0.67** |  |  |  |  |  |
| 2 | Frightful |  |  | **0.71** |  |  |  |  |
| 3 | Lonely | **0.74** |  |  |  |  |  |  |
| 4 | Not strong | 0.37 | 0.37 |  | 0.42 |  |  |  |
| 5 | Not confident |  | 0.47 | 0.35 | 0.46 |  |  |  |
| 6 | Afraid |  |  | **0.77** |  |  |  |  |
| 7 | Deserted | **0.85** |  |  |  |  |  |  |
| 8 | Weak | 0.49 |  |  | 0.38 |  |  |  |
| 9 | Not safe | 0.36 |  |  | 0.44 |  |  |  |
| 10 | Not independent |  |  |  | **0.70** |  |  |  |
| 11 | Desolate | **0.68** |  |  |  |  |  |  |
| 12 | Tense |  |  | **0.72** |  |  |  |  |
| 13 | Not glad |  | **0.80** |  |  |  |  |  |
| 14 | Not proud |  | **0.80** |  |  |  |  |  |
| 15 | Abandoned | **0.82** |  |  |  |  |  |  |
| 16 | Not composed |  |  |  | **0.71** |  |  |  |
| 17 | Not relaxed |  |  | 0.40 | **0.64** |  |  |  |
| 18 | Not happy |  | **0.85** |  |  |  |  |  |
| 24 | Pain |  |  | **0.51** |  |  |  | 0.33 |
| 19 | Panic |  |  | **0.65** |  |  |  |  |
| 20 | Hopelessness | 0.47 |  | 0.46 |  |  |  |  |
| 21 | Not longing for the child |  | 0.32 | -0.30 |  | 0.31 |  |  |
| 22 | No self confidence |  | **0.54** |  | 0.32 |  |  |  |
| 23 | No trust | 0.**61** |  |  |  |  |  |  |
| 25 | Behave badly |  |  |  |  |  |  | **0.75** |
| 26 | Not let happen |  |  |  |  | 0.40 |  | -0.40 |
| 27 | Lose control |  |  |  |  |  |  | **0.82** |
| 28 | Not joyful |  |  |  |  | **0.68** |  |  |
| 29 | Not natural |  |  |  |  | **0.80** |  |  |
| 30 | Not obvious |  |  |  |  | **0.80** |  |  |
| 31 | Dangerous |  |  |  |  | 0.35 | 0.45 |  |
| 32 | Fantasies that child will die |  |  |  |  |  | **0.89** |  |
| 33 | Fantasies that child will be injured |  |  |  |  |  | **0.87** |  |

Comment: only factor loading > 0.29 are presented. Factor loading > 0.50 are bold.
